# Third-trimester Reference Ranges for Cerebroplacental Ratio and Pulsatility Index for Middle Cerebral Artery and Umbilical Artery in Normal-growth Singleton Fetuses in the Israeli Population

**DOI:** 10.5041/RMMJ.10379

**Published:** 2019-10-29

**Authors:** Efraim Zohav, Eyal Zohav, Mark Rabinovich, Ahmad Alasbah, Simon Shenhav, Hadar Sofer, Yaniv S. Ovadia, Eyal Y. Anteby, Leonti Grin

**Affiliations:** 1 Ultrasound unit, Department of Obstetrics and Gynecology, Barzilai University Medical Center, Faculty of Health Sciences, Ben-Gurion University of the Negev, Israel; 2 Lis Maternity & Women’s Hospital–Tel Aviv Sourasky Medical Center, Tel Aviv University, Tel Aviv, Israel

**Keywords:** Cerebroplacental ratio, fetal Doppler, middle cerebral artery, umbilical artery

## Abstract

**Background:**

The ratio between the fetal umbilical artery pulsatility index (UA-PI) and the middle cerebral artery pulsatility index (MCA-PI) is termed the cerebroplacental ratio (CPR). The CPR represents fetal blood flow redistribution at the early stages of placental insufficiency; moreover, it has predictive value for adverse intrapartum and neonatal outcomes. However, internationally accepted reference ranges for CPR are lacking.

**Objective:**

This study sought to establish UA-PI, MCA-PI, and CPR reference ranges in low-risk, normal-growth singleton fetuses during the third trimester of pregnancy.

**Methods:**

A retrospective cohort cross-sectional study was performed in the obstetrics ultrasound unit of a university hospital in Israel. We reviewed all fetal and maternal electronic records of pregnant women referred for ultrasound assessment during the third trimester between January 2014 and January 2019. We included only singleton pregnancies with normal anatomy scans and a normal third-trimester estimated fetal weight. The UA-PI, MCA-PI, and CPR reference ranges were reconstructed for each of the vessels for each gestational age between 29 and 41 weeks.

**Results:**

A total of 560 pregnancies met the inclusion criteria. Satisfactory waveforms and measurements were obtained in all cases. At least 18 women enrolled at each gestational week. The MCA-PI and CPR values showed a similar parabolic curve during the third trimester of pregnancy, with a peak value at 32 and 33 gestational weeks, respectively. The UA-PI showed a linear and gradual decrease over the gestational age.

**Conclusions:**

In this study we established UA-PI, MCA-PI, and CPR reference ranges in low-risk, normal-growth singleton fetuses during the third trimester based on the Israeli population.

## INTRODUCTION

Fetal circulation has been studied extensively in the last decades. Knowledge of normal fetal blood flow in fetuses with adequate growth for gestational age is essential for early diagnosis of a pathological condition that could place fetal well-being at risk, and to prevent fetal morbidity and mortality. Fetal adequate blood flow circulation is mainly dependent on normal placental anatomy and development during pregnancy. An abnormal or malfunctioning placenta can directly affect fetal circulation. The umbilical artery (UA) is usually the first fetal blood vessel to be affected by placental insufficiency. The initial increase in placental blood flow, vascular impedance, causes a retrograde increase of blood flow resistance in the UA.^1^ When placental insufficiency further deteriorates, blood flow resistance in the descending aorta increases, resulting in more blood diverted through the aortic isthmus shunt to reach the fetal brain. This phenomenon is reflected by a decreased middle cerebral artery pulsatility index (MCA-PI), making it the second vascular marker in the cascade of placental insufficiency.^2^,^3^ Correct diagnoses of early signs of placental insufficiency through fetal blood flow redistribution has been studied extensively in the literature.^4^,^5^ Early detection of abnormal blood flow redistribution patterns is important for efficient fetal Doppler monitoring and is part of meticulous surveillance, with a potential benefit to reduce fetal morbidity and mortality.^6^–^8^ Cerebroplacental ratio (CPR) has been studied and suggested as the most efficient vascular index to detect the above-mentioned fetal redistribution patterns.^9^–^12^ Efficient detection of fetal blood flow redistribution as a predictor of adverse perinatal outcome requires knowledge on the threshold value of CPR, to determine if it is pathologic or normal. Previous studies found a fixed cut-off for the CPR value in pregnancy.^13^,^14^ Other studies established CPR reference ranges in their healthy pregnant mothers and fetal population, and defined the weekly CPR value below the 10th or 5th percentile as a threshold cut-off indicator of pathology.^5^,^15^ Many CPR reference ranges have been published from different geographic regions. A recent meta-analysis found considerable variation between these CPR reference ranges, which has important implications for clinical practice.^16^

Hitherto, the umbilical artery pulsatility index (UA-PI), MCA-PI, and CPR reference ranges based on examinations in the population of Israel have not yet been established, and local clinical practice defaults rely on external reference range threshold values to classify the fetal population as either normal or abnormal.^14^,^17^ Therefore, the present study, performed in Israel, aimed to establish UA-PI, MCA-PI, and CPR reference range percentiles and standard deviation (SD) in healthy, low-risk, normal-growth singleton fetuses during the third trimester of pregnancy.

## METHODS

### Settings

This was a retrospective cohort cross-sectional study in the obstetrics ultrasound unit of Barzilai University Medical Center, in Ashkelon, Israel. This tertiary referral center serves about half a million residents in a large region of southwest Israel, with nearly 5,000 births annually.

### Participants

The study was approved by the ethics committee of Barzilai University Medical Center. After establishment of a comprehensive database from the ultrasound unit and delivery room, we reviewed all fetal and maternal electronic records in all consecutive pregnant women with singleton fetuses and CPR measurements, who were referred for fetal growth ultrasound assessment between January 2014 and January 2019. Inclusion criteria for the study were singleton pregnancies with normal second-trimester anatomy, normal term estimated fetal weight, and birth weights between the 10th and 90th percentiles.^4^ Gestational age based on the last menstrual period (LMP) date was corrected if first-trimester fetal sonographic age differed from the LMP date by 4 days or more. Exclusion criteria were: pregnancies complicated by maternal diseases such as chronic hypertension, preeclampsia, and diabetes.

### Assays

Measurements were performed in the third trimester of pregnancy between 29 to 42 weeks, the period that CPR measurements are relevant for the diagnosis, follow-up, and management of intrauterine growth restriction.

The CPR was calculated as the ratio between the MCA-PI and UA-PI. Sonographic Doppler examinations and fetal biometry were performed by sonographers experienced in Doppler examination, based on fetal Doppler criteria measurements of MCA and UA according to the International Society of Ultrasound in Obstetrics and Gynecology (ISUOG) published in 2013,^18^ before the dataset of the current study was created. A pulsed-wave Doppler gate was placed at the proximal third of the MCA, close to its origin in the internal carotid artery. The angle between the ultrasound beam and the direction of blood flow was kept as close as possible to 0 degrees and no more than 15 degrees. At least three and fewer than 10 consecutive waveforms were recorded. Caliper lines were placed inside the border of the vessel. Umbilical artery Doppler measurements were performed in a non-moving free loop when at least three consecutive uniform flow velocity waveforms with a high signal-to-noise ratio were obtained.^19^,^20^ All scans were performed using an abdominal RAB 4–8D probe of ultrasound model (Voluson E6, General Electric Company, Zipf, Austria). Only the last CPR examination of each pregnancy was based on the Hadlock formula and included four fetal organ measurements: head circumference, biparietal diameter, abdominal diameter, and femur length.^21^ The estimated fetal weight percentiles were based on recently published new Israeli fetal weight curve reference ranges.^22^ Birth weight percentiles were based on Israeli live-born birth weight standards.^23^

### Data and Statistical Analysis

All statistical analyses were performed with software package JMP (version 14.2, SAS Institute Inc., Cary, NC, USA). Patient demographics and obstetrical characteristics are given as mean and standard deviation (SD) as well as the median and interquartile range (represented by the 25th and 75th percentiles). Categorical data are given as a percentage of the total. Sonographic estimation of Doppler indices, the UA-PI, MCA-PI, and CPR are given as mean, median, SD, and 5th, 10th, 25th, and 75th percentiles for every gestational age.

## RESULTS

A total of 560 pregnant women met the inclusion criteria and were enrolled in the study. Satisfactory waveforms and measurements were obtained in all cases. The demographic and obstetric variables of the study group are shown in Table 1. At least 18 women at each gestational week were included in the study. The mean and median values, as well as 5th, 10th, 25th, and 75th percentiles of the UA-PI, MCA-PI, and CPR reference ranges are shown in Tables 2, 3, and 4, respectively.

**Table 1:** Demographic and Obstetrics Study (*n*=560).

Demographic and Obstetric Variables	Mean±SD*, Median (25th–75th percentile range)^†^, or %^‡^
Maternal age (years)	30 (27–34)^†^
Body mass index (kg/m^2^)	27.7±4.8*
Gravidity (*n*)	2 (1–3)^†^
Parity (*n*)	2 (1–3)^†^
Birth weight (g)	2900 (2630–3120)^†^
Birth weight percentile (%)	26 (15–40)^†^
Gestational age at examination (weeks)	37.3±2.6*
Fetal sex: female (%)	51.32^‡^
Fetal sex: male (%)	48.68^‡^
Primigravida (%)	30.71^‡^
Multigravida (%)	69.29^‡^

**Table 2:** Cerebroplacental Ratio (CPR), Number of Exams (*n*), Percentiles, Mean, Median, Standard Deviation (SD), and Minimum (Min) and Maximum (Max) Values.

Gestational Age (wk)	Cerebroplacental Ratio Percentiles										
	*n*	% of Total	Min	5th	10th	25th	Median	Mean	SD	75th	Max
29	22	3.92%	0.90	0.92	1.04	1.30	1.80	1.82	0.67	2.13	3.64
30	23	4.18%	0.70	0.72	0.83	1.50	1.80	1.93	0.83	2.50	4.42
31	18	5.53%	1.00	1.00	1.17	1.50	1.98	1.92	0.51	2.40	2.63
32	29	5.71%	1.00	1.09	1.30	1.60	2.10	2.20	0.73	2.72	3.61
33	27	4.82%	1.30	1.30	1.30	1.60	2.20	2.27	0.75	2.90	3.70
34	40	7.14%	0.80	1.01	1.12	1.34	2.03	2.00	0.69	2.50	3.36
35	52	9.28%	1.06	1.09	1.40	1.70	2.03	2.10	0.59	2.50	4.48
36	68	12.08%	0.70	0.98	1.10	1.50	1.80	1.89	0.64	2.29	4.50
37	93	16.92%	0.74	1.03	1.13	1.46	1.80	1.93	0.73	2.23	5.60
38	76	13.57%	0.68	1.09	1.10	1.39	1.68	1.77	0.55	2.10	3.38
39	61	10.91%	0.78	0.89	1.01	1.30	1.64	1.63	0.45	1.96	2.80
40	32	5.75%	1.05	1.08	1.14	1.32	1.61	1.71	0.49	1.95	2.90
41	19	3.35%	0.60	0.60	1.00	1.46	1.85	1.71	0.50	2.10	2.70

**Table 3:** Middle Cerebral Artery Pulsatility Index (MCA-PI), Number of Exams, Percentiles, Mean, Median, Standard Deviation (SD), and Minimum (Min) and Maximum (Max) Values.

Gestational Age (wk)	Middle Cerebral Artery Pulsatility Index Percentiles										
	*n*	% of Total	Min	5th	10th	25th	Median	Mean	SD	75th	Max
29	22	3.92%	0.85	0.85	1.19	1.53	2.05	2.07	0.67	2.50	3.49
30	23	4.18%	1.29	1.29	1.35	1.59	2.00	2.06	0.63	2.19	3.60
31	18	5.53%	1.40	1.40	1.46	1.64	1.96	2.08	0.58	2.39	3.29
32	29	5.71%	1.30	1.30	1.35	1.80	2.24	2.28	0.66	2.61	4.00
33	27	4.82%	1.28	1.32	1.50	1.87	2.00	2.13	0.49	2.50	3.06
34	40	7.14%	1.10	1.24	1.37	1.58	1.90	1.91	0.47	2.18	3.20
35	52	9.28%	1.28	1.37	1.44	1.67	1.89	1.98	0.48	2.20	3.99
36	68	12.08%	1.00	1.13	1.22	1.56	1.77	1.78	0.37	2.00	2.60
37	93	16.92%	0.99	1.04	1.15	1.38	1.65	1.74	0.55	1.97	4.10
38	76	13.57%	0.73	0.86	1.02	1.31	1.52	1.55	0.43	1.77	3.20
39	61	10.91%	0.72	0.90	1.03	1.22	1.40	1.40	0.28	1.55	2.13
40	32	5.75%	0.87	0.88	1.00	1.14	1.28	1.33	0.32	1.38	2.20
41	19	3.35%	0.66	0.66	1.03	1.27	1.42	1.44	0.34	1.74	2.10

**Table 4:** Umbilical Artery Pulsatility Index (UA-PI), Number of Exams (*n*), Percentiles, Mean, Median, Standard Deviation (SD), and Minimum (Min) and Maximum (Max) Values.

Gestational Age (wk)	Umbilical Artery Pulsatility Index Reference Ranges Value										
	*n*	% of Total	Min	5th	10th	25th	Median	Mean	SD	75th	Max
29	22	3.92%	0.74	0.74	0.80	0.99	1.22	1.21	0.26	1.37	1.85
30	23	4.18%	0.70	0.70	0.78	0.95	1.17	1.20	0.34	1.27	2.16
31	18	5.53%	0.60	0.60	0.65	0.91	1.01	1.04	0.21	1.24	1.31
32	29	5.71%	0.83	0.84	0.88	0.93	1.02	1.03	0.12	1.14	1.27
33	27	4.82%	0.68	0.69	0.74	0.80	0.98	0.98	0.19	1.11	1.44
34	40	7.14%	0.59	0.67	0.77	0.88	1.02	1.02	0.19	1.17	1.38
35	52	9.28%	0.63	0.67	0.76	0.83	0.90	0.95	0.18	1.05	1.42
36	68	12.08%	0.36	0.64	0.68	0.87	0.99	0.99	0.23	1.15	1.72
37	93	16.92%	0.52	0.63	0.70	0.81	0.94	0.94	0.18	1.05	1.36
38	76	13.57%	0.60	0.66	0.68	0.80	0.91	0.93	0.22	1.00	2.00
39	61	10.91%	0.58	0.63	0.69	0.74	0.83	0.87	0.16	0.98	1.33
40	32	5.75%	0.53	0.57	0.63	0.69	0.77	0.80	0.16	0.91	1.14
41	19	3.35%	0.58	0.58	0.63	0.69	0.81	0.90	0.41	0.91	2.33

Median and mean values of UA-PI steadily decreased in a linear correlation with increase in gestational age. The MCA-PI reference ranges have a parabolic shape curve with a peak at 32 to 33 weeks. The CPR values demonstrated a parabolic curve similar to the MCA-PI curve with the mean peak value at 33 and 32 gestational weeks, respectively.

**Figure 1:**
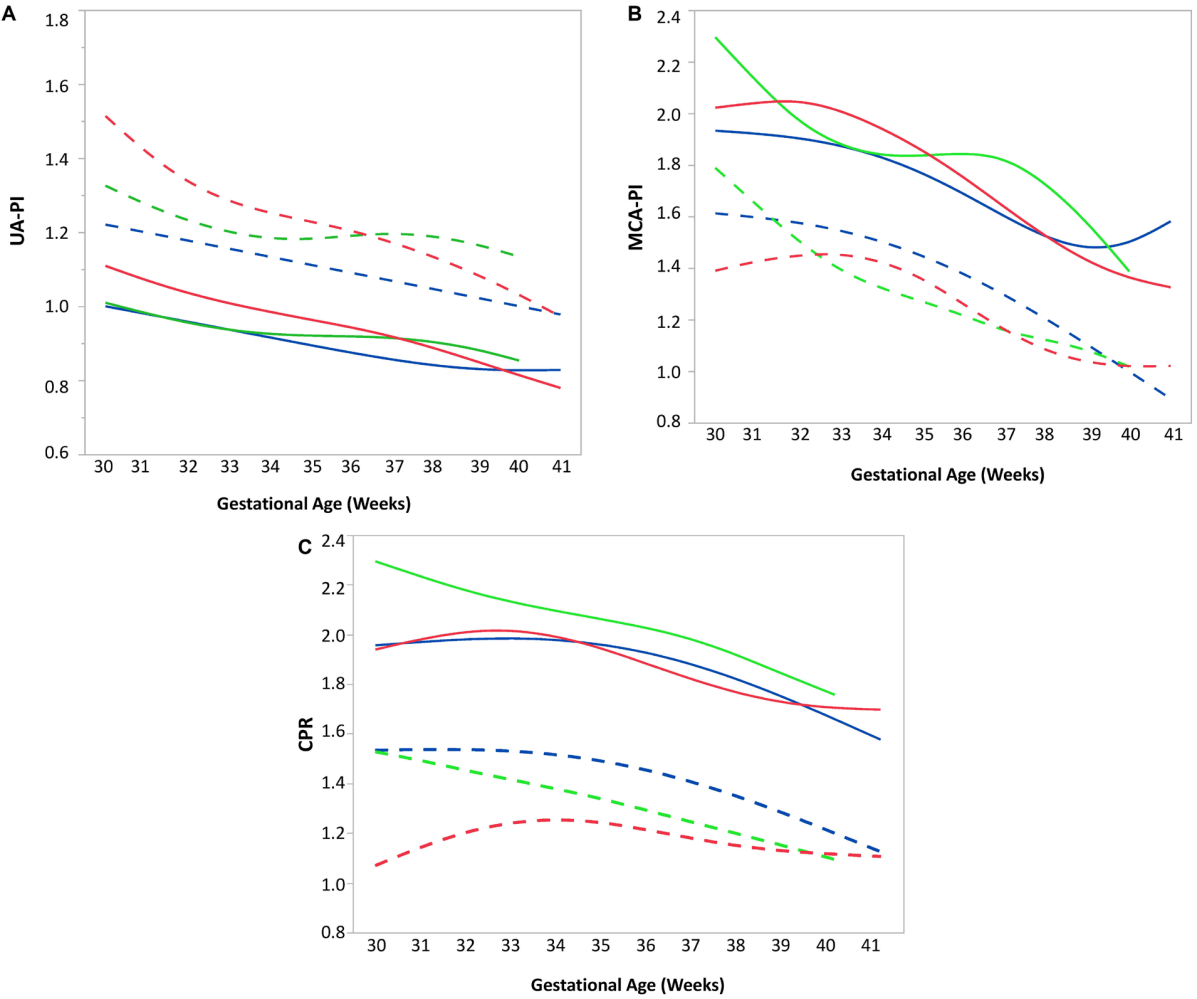
Comparison of Doppler Studies Between Israeli and International Reference Curves. **A:** Comparison of 50th (solid lines) and 90th (dashed lines) percentiles of umbilical artery pulsatility index (UA-PI). **B:** Comparison of 50th (solid lines) and 10th (dashed lines) percentiles of middle cerebral artery pulsatility index (MCA-PI). **C:** Comparison of cerebroplacental ratio (CPR), 50th (solid lines) and 10th (dashed lines) according to gestational age. **Panels A–C:** Red dashed and solid lines: Barzilai Medical Center local data; green dashed and solid lines: based on data from Baschat et al.^5^; blue dashed and solid lines: based on data from Ciobanu et al.^24^.

In Figure 1 we compared two international studies^5^,^24^ with our data from local curves for UA-PI, MCA-PI, and CPR.

The median and 90th percentile of the UA-PI was higher in our group compared to the international references, until 36 weeks. The shape of the median and 10th percentile of the MCA-PI follows a similar pattern to Ciobanu et al.^24^ and differs from Baschat et al.^5^

Our median CPR curve shape is similar to the values of Ciobanu et al. throughout the whole period and lower than the median of Baschat et al.

## DISCUSSION

This is the first study that established UA-PI, MCA-PI, and CPR reference range percentiles in low-risk, unselected, normal-growth singleton fetuses during the third trimester of pregnancy in Israel. Based on our study population, the mean MCA-PI and mean CPR increased with gestational age, reaching a peak at approximately 32 and 33 weeks, respectively, whereas the UA-PI decreased linearly with gestation. Our results are generally in line with previously published CPR reference range curves^5^,^24^ with some differences, as shown in Figure 1.

Among the available parameters in the relevant literature, CPR based on pulsatility index (PI) was found to be the most accurate predictor for fetal distress antepartum.^9^,^11^,^12^,^25^ When using PI in CPR, the formula of PI (peak systolic velocity minus end diastolic velocity divided by the mean value) can yield a wider range of waveform value patterns and provides fine accuracy of vascular resistance. Therefore, PI was included in all reference ranges established in the current study.

The CPR reflects fetal blood flow redistribution during the early stages of placental insufficiency. It is generally accepted that a pathologic CPR during the third trimester should prompt closer monitoring of the fetus with a repeat Doppler study. The use of vascular markers is well accepted in order to prevent further deterioration which may, in turn, result in severe fetal morbidity and mortality. The decision to deliver the fetus during the preterm period is mainly based on more severe changes in the umbilical artery blood flow, when the increase in placental vessel blood flow resistance causes the absence of umbilical artery end-diastolic flow.^26^ In this severe situation, CPR does not play a role in the decision to deliver the fetus prematurely. The CPR is an efficient marker both for the detection and follow-up of early-stage placental insufficiency during the late preterm period.^11^ The reconstructed CPR reference ranges are based on healthy normal-growth fetuses during the period of clinical relevance from 29 to 41 weeks of gestation. The UA-PI values showed a gradual and steady decline with advancing gestational age. The MCA-PI and CPR values showed a parabolic pattern, with the mean peak value at 32 and 33 weeks of gestation, respectively.

The steady decrease in UA-PI represents a process of placental adaptation to the increasing fetal needs for oxygen supply with a steady decrease in blood vessel resistance during the third trimester of pregnancy. The parabolic pattern curve of MCA-PI represents the increasing gap between the placental growth and fetal blood supply that emerges during late preterm and term pregnancy. The similarities and differences between different CPR references have been previously discussed in the literature.^16^,^24^ As with fetal weight estimation, CPR values may also be affected by the geographic locale, environment, and clinical setting. The exact etiology for the observed differences is still unclear. However, CPR is a valuable clinical tool in the obstetrician’s armamentarium in pregnancy surveillance. While there is no internationally accepted reference as the gold standard for CPR values, centers should consider the possible advantages of creating local references based on their population of patients.

## CONCLUSIONS

This is the first study to establish UA-PI, MCA-PI, and CPR reference range percentile tables during the third trimester of pregnancy based on the Israeli population. The present findings have an important implication on Doppler surveillance in low-risk pregnancies with pathological CPR in the third trimester of pregnancy. Future prospective studies are needed to validate these reference range tables.

## LIMITATIONS

This study was limited by its retrospective cohort study data. Given the small number of included cases between 38–40 weeks of gestation, the results of our Doppler measurements for this gestation period should be taken with caution.

## Abbreviations

CPR: cerebroplacental ratio
LMP: last menstrual period
MCA-PI: middle cerebral artery pulsatility index
PI: pulsatility index
SD: standard deviation
UA: umbilical artery
UA-PI: umbilical artery pulsatility index.
